# Two new species of *Anatkina* (Hemiptera, Cicadellidae, Cicadellinae) from China

**DOI:** 10.3897/zookeys.1273.175505

**Published:** 2026-03-23

**Authors:** Li-kun Zhong, Yan Jiang, Xi Chen, Mao-fa Yang

**Affiliations:** 1 Institute of Entomology, Guizhou Key Laboratory of Agricultural Biosecurity, Guizhou University, Guiyang 550025, China Institute of Entomology, Guizhou Key Laboratory of Agricultural Biosecurity, Guizhou University Guiyang China; 2 Department of Pharmacy, Guizhou Provincial Engineering Research Center of Medical Resourceful Healthcare Products, Guiyang Healthcare Vocational University, Guiyang 550000, China College of Tobacco Sciences, Guizhou University Guiyang China; 3 Guiyang Grain for Green Program Service Center, Guiyang 550002, China Department of Pharmacy, Guizhou Provincial Engineering Research Center of Medical Resourceful Healthcare Products, Guiyang Healthcare Vocational University Guiyang China; 4 College of Tobacco Sciences, Guizhou University, Guiyang 550025, China Guiyang Grain for Green Program Service Center Guiyang China

**Keywords:** Auchenorrhyncha, Hemiptera, identification key, leafhopper, morphology, taxonomy

## Abstract

Two new species of the genus *Anatkina* Young, 1986 from China are described and illustrated: *Anatkina tortuosa* Zhong & Yang, **sp. nov**. and *Anatkina maolanensis* Zhong & Yang, **sp. nov**. Voucher specimens of the new taxon are curated at the Institute of Entomology, Guizhou University, Guiyang, China (GUGC). A revised key to *Anatkina* species from China is also provided.

## Introduction

The genus *Anatkina* currently comprises 59 valid species and is widely distributed throughout South and Southeast Asia, including India, Thailand, Vietnam, Indonesia, Myanmar, the Philippines, China, and adjacent regions (Yang et al. 2017). Among these areas, southwestern China (Sichuan, Guizhou, Yunnan, and Xizang) constitutes a major center of species diversity within the family Cicadellidae. This remarkable diversity is primarily attributed to the region’s complex topography, characterized by steep elevational gradients and numerous intermontane basins. Notably, 25 of the 36 valid Chinese species of *Anatkina* have been recorded in this region (Yang and Li 1998, 1999, 2001, 2005; Yang and Zhang 2000; Yang et al. 2001; Yang et al. 2015). In the present paper, two new species of *Anatkina* from southwestern China, *A. tortuosa* sp. nov. and *A. maolanensis* sp. nov., are described and illustrated. Comprehensive morphological descriptions, including details of the male genitalia and habitus photographs, are provided. Furthermore, an updated key to all known *Anatkina* species from China is presented to facilitate future taxonomic work.

## Material and methods

Specimens were collected during the daytime by sweeping over shrubs and weeds with insect nets, and at dusk (18:00–23:00) using high-pressure mercury lamps. All specimens were preserved in absolute ethanol and stored at -20 °C. The abdomens were dissected and transferred to glycerol for detailed observation and imaging. Subsequently, they were permanently stored in glycerol within microcentrifuge tubes. The habitus and male genitalia were photographed using a KEYENCE VHX-6000 digital imaging system and a Nikon Eclipse Ni-E microscope, respectively. Image compositing and optimization were carried out using Adobe Photoshop 2020. The holotypes of *A. tortuosa* sp. nov. and *A. maolanensis* sp. nov. are deposited in the Institute of Entomology, Guizhou University, Guiyang, China (GUGC). Morphological terminology follows Young (1986) and Dietrich (2005).

## Taxonomy

### Genus Anatkina

Taxon classificationAnimaliaHemipteraCicadellidae

Young, 1986

58DBFDEC-61CC-5EEA-8265-8ACB7D04C52D

Anatkina Young, 1986: 39

#### Type species.

*Tettigonia vespertinula* Breddin, 1903.

#### Distribution.

Oriental.

### Anatkina tortuosa

Taxon classificationAnimaliaHemipteraCicadellidae

Zhong & Yang sp. nov.

DC9B5C6B-FC1F-5B28-B029-F8C8D22C3BC8

https://zoobank.org/F3629A0B-D949-42C5-B525-0E91CF732C77

Figs 1A–D, 2A–F

#### Material examined.

***Holotype***: • ♂, Motuo County, Xizang Autonomous Region, China, 18 August 2020, coll. Xian-Yi Wang.

**Figure 1.**
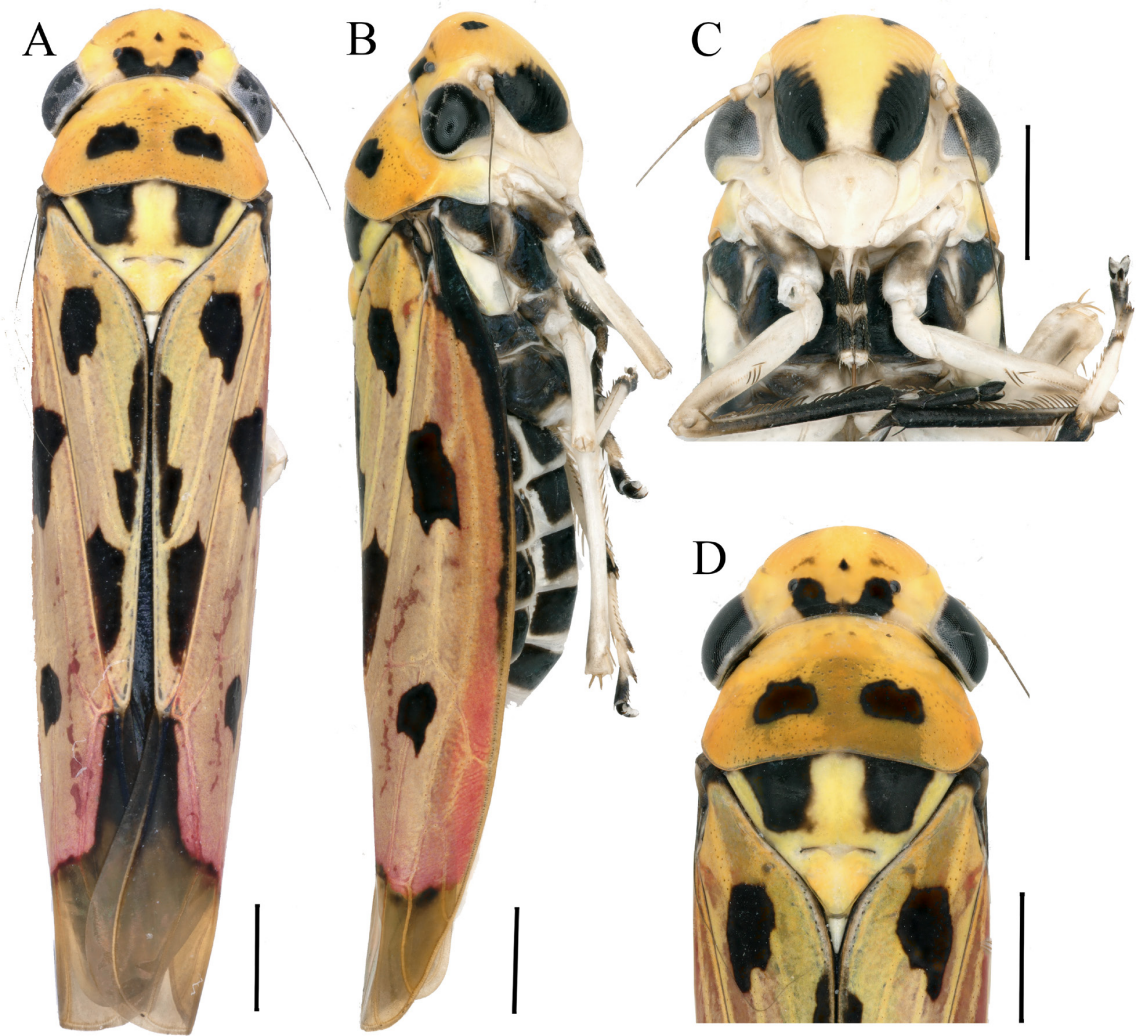
External morphology of *Anatkina tortuosa* Zhong & Yang, sp. nov., male holotype. **A**. Habitus, dorsal view; **B**. Habitus, lateral view; **C**. Face, anterior view; **D**. Head and pronotum, dorsal view. Scale bars: 1000 μm.

**Figure 2.**
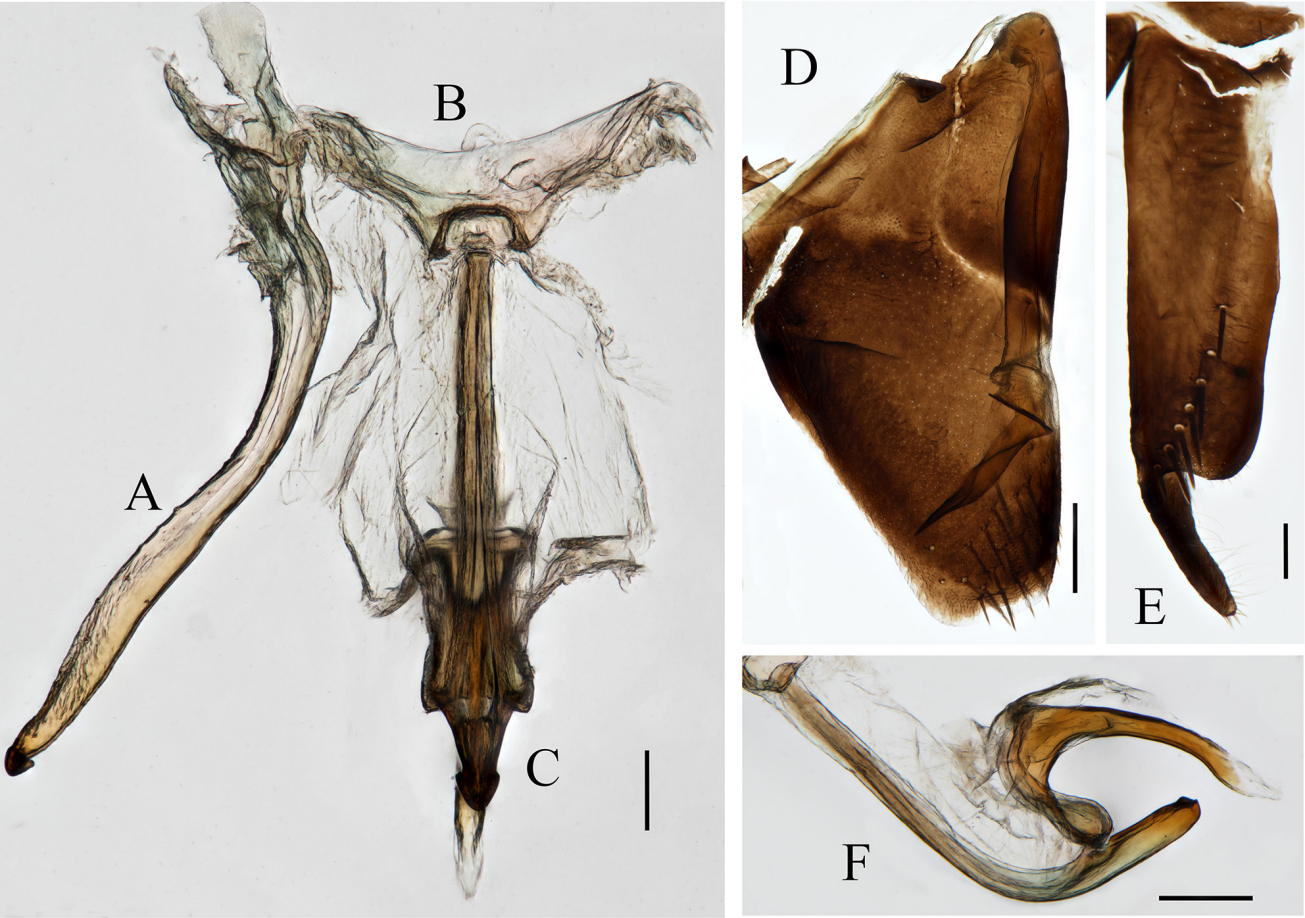
Male genitalia of *Anatkina tortuosa* Zhong & Yang, sp. nov. **A**. Style; **B**. Connective; **C**. Aedeagus and paraphysis, ventral view; **D**. Pygofer and pygofer process, lateral view; **E**. Subgenital plate, ventral view; **F**. Aedeagus and paraphysis, lateral view. Scale bars: 200 μm.

#### Description.

Length of male 9.6 mm.

Head and thorax orange-yellow in dorsal view. Face yellowish-white, frontoclypeus with large black macula on each side, anteclypeus ivory. Head vertex with two rhomboid black spots; crown bearing a large U-shaped black spot extending from the base to the ocelli, and three faint black maculae anterior to the ocelli. Ocelli, eyes and antennae gray. Pronotum exhibits paired medial black maculae against a densely punctate grey background. Scutellum with black spots bordering the anterior margin near each basal angle; apical angle grayish-white. Thoracic sternum black with ivory stripes, legs yellowish-white, tibiae of prothoracic legs, tarsi and pretarsi all black. Abdomen black in ventral view, sternites with posterior and lateral margins pale. Forewing shading from yellowish-white basally to light coral apically, with the membranous apex blackish-brown and bearing eight black markings: one spot at base, one longitudinal dagger-shaped stripe at median portion of clavus near posterior margin of forewing, two large spots near base and apex of clavus respectively, two large spots at one-third and two-thirds of corium, and a single stripe traverses the subapical forewing, crossing all apical cells and terminating at the first apical cell.

Head moderately produced, anterior margin broadly round, median length of crown 1/2 of interocular width, coronal suture distinct only at base of crown, ocelli located slightly behind imaginary line between anterior eye angles, lateral frontal sutures extending onto crown and attaining ocelli, frontoclypeus nearly flattened medially, muscle impressions distinct, transclypeal suture complete. Pronotum width equal to transocular width of head, anterior margin rounded, posterior margin shallowly concave medially, disk with minutely punctuate at median and posterior portion. Scutellum slightly convex behind transverse depression. Forewing with apical membrane distinct, base of second apical cell almost aligned with base of third.

Pygofer short, in lateral view with posterior margin subtruncate, surface with macrosetae on posterior margin. Pygofer process incurved, its apical one-third twisted and curved posterodorsally, falling short of the pygofer margin, apex acute. Subgenital plates slender, produced posteriorly farther than pygofer, abruptly narrowed on apical one-four portion and curved dorsad, apical half of surface with longitudinal uniseriate macrosetae medially and short microsetae. Aedeagus with preatrium curved posteroventrad and articulated with paraphyses, shaft slightly curved ventrally, extending posteriorly beyond apex of paraphyses, apex rounded. Paraphyses slender, with posterior one-third curved dorsally, apex corniculate projections. Connective broadly U-shaped. Style slender, apical portion abruptly narrowed, with hooked apex.

#### Distribution.

China (Xizang).

#### Etymology.

The specific epithet “tortuosa”, a Latin term meaning “twisted” or “convoluted”, directly characterizes the contorted morphology of the pygofer process.

#### Remarks.

This species is similar to *A. illustris* (Distant, 1908), *A. incurvata* Kuoh, 1991, *A. nigriventris* Li, 1992, *A. zhoui* Yang & Li, 2001, *A. jianfengana* Yang & Li, 2001, and *A. leishanensis* Yang, Meng & Yu, 2015 in appearance, but differs from them by the following male genitalic characters: (1) pygofer process incurved, its apical one-third twisted and curved posterodorsally, (2) subgenital plates slender, produced posteriorly farther than pygofer, abruptly narrowed on apical one-four portion and curved dorsad, and (3) style slender, apical portion abruptly narrowed, with a hooked and acute apex.

### Anatkina maolanensis

Taxon classificationAnimaliaHemipteraCicadellidae

Zhong & Yang sp. nov.

64587F65-F6A5-54CA-9156-6F584D6C0486

https://zoobank.org/4018A3E3-CDC0-46CB-B524-C322CCB12E65

Figs 3A–D, 4A–F

#### Material examined.

***Holotype***: • ♂, Maolan National Nature Reserve, Guizhou, China, 13 April 2019, coll. Yan Jiang.

**Figure 3.**
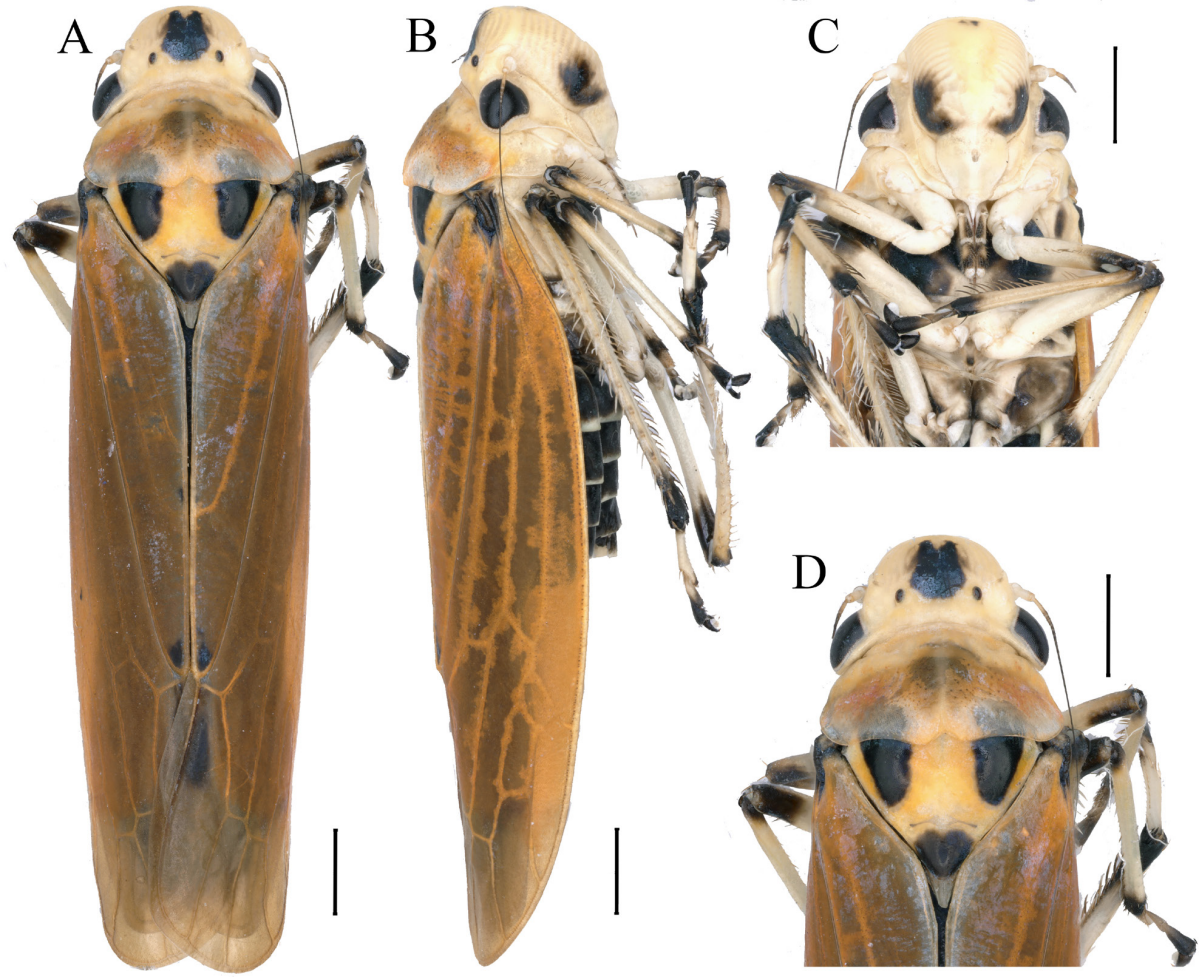
External morphology of *Anatkina maolanensis* Zhong & Yang, sp. nov., male holotype. **A**. Habitus, dorsal view; **B**. Habitus, lateral view; **C**. Face, anterior view; **D**. Head and pronotum, dorsal view. Scale bars: 1000 μm.

**Figure 4.**
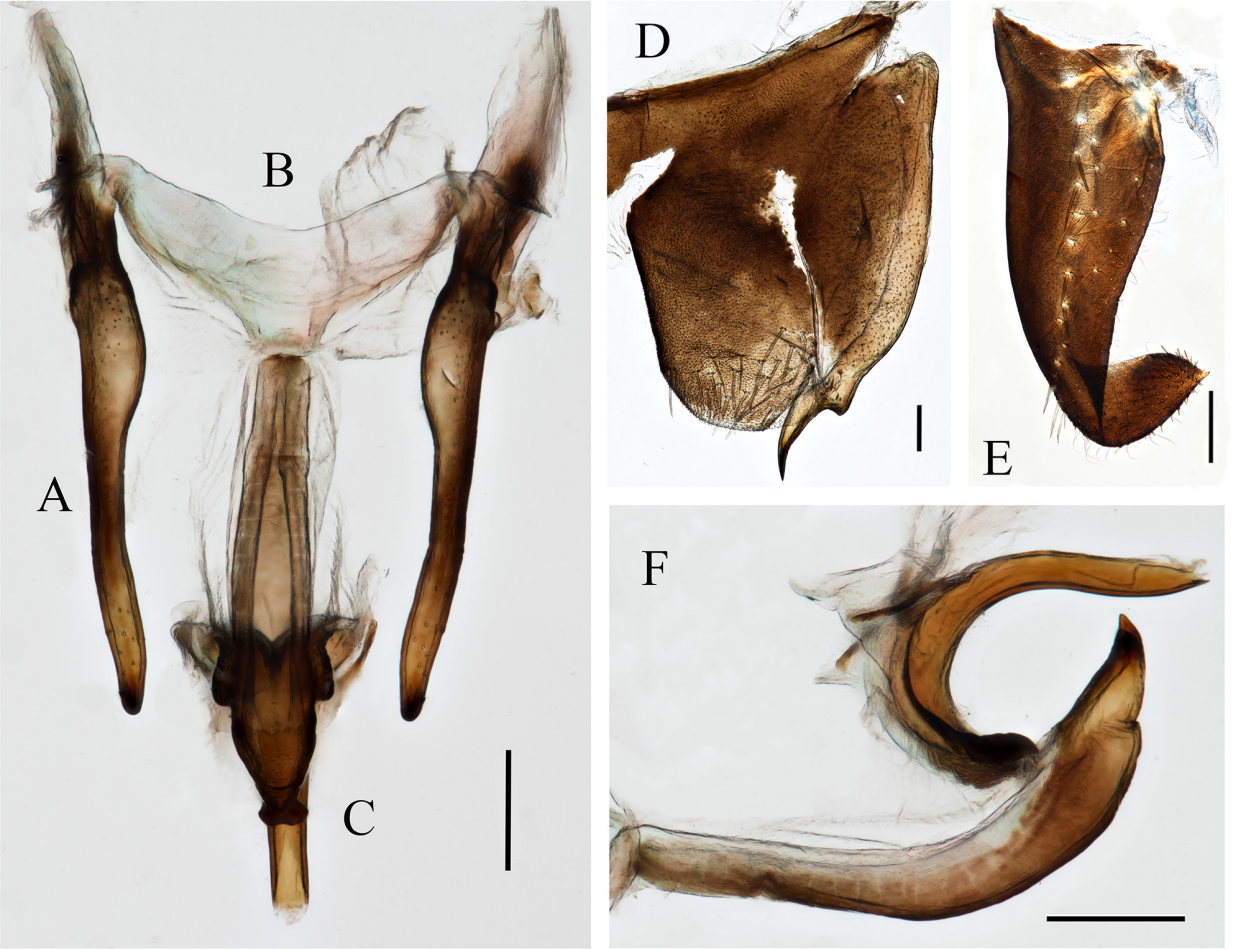
Male genitalia of *Anatkina maolanensis* Zhong & Yang, sp. nov. **A**. Style; **B**. Connective; **C**. Aedeagus and paraphysis, ventral view; **D**. Pygofer and pygofer process, lateral view; **E**. Subgenital plate, ventral view; **F**. Aedeagus and paraphysis, lateral view. Scale bars: 200 μm.

#### Description.

Length of male 10.8 mm.

Head and thorax yellowish-white in dorsal view. Face yellowish-white, frontoclypeus with large black macula on each side, anteclypeus ivory. Head vertex with one elliptical black spot. Crown with a large bipartite black macula anteromedially between the ocelli. Ocelli, inner margin of eyes, antennae and pronotum yellowish-brown. Scutellum with paired anterolateral black spot near the basal angles and an apical black spot. Thoracic sternum black with ivory stripes, legs yellowish-white, femora, tibiae and tarsi apex black. Abdomen black in ventral view, sternites with posterior and lateral margins pale. Forewing yellowish-brown in dorsal view, clavus with a large black macula basally and a small black macula apically.

Head prominently produced, anterior margin broadly round, median length of crown 2/3 of interocular width, ocelli located slightly front imaginary line between anterior eye angles, lateral frontal sutures extending onto crown and attaining ocelli. Frontoclypeus slightly convex medially, muscle impressions distinct, transclypeal suture complete. Pronotum broader than head, anterior margin rounded, posterior margin wavy and concave medially, disk with minutely punctuate at median portion. Scutellum slightly convex behind transverse depression. Forewing with apical membrane indistinct, base of second apical cell slightly more proximal than base of third.

Pygofer produced, in lateral view with posterior margin roundly convex, surface with macrosetae on posterior margin. Pygofer process extending posteriorly farther than pygofer apical margin, apical one-fifth portion bent posteroventrally and with acute apex, dorsal margin with minute denticles subapically. Subgenital plates short, progressively narrower toward apical portion, apical portion one-fourth dorsally curved, surface with longitudinal uniseriate macrosetae and few short microsetae on inner posterolateral margin, apical spine. Aedeagus with preatrium curved posteroventrad, articulating with paraphyses, shaft extending posteriorly beyond apex of paraphyses. Paraphyses slender, in lateral view bent dorsally at middle, apex corniculate projections laterally on both sides. Connective V-shaped. Style almost straight, curved apically, apex rounded.

#### Distribution.

China (Guizhou).

#### Etymology.

The new species name refers to the type locality, the Maolan National Nature Reserve.

#### Remarks.

This species is similar to *A. zuoi* Yang & Zhang, 2000, *A. nigrilega* Yang, Meng & Li, 2017 in appearance, but it is unambiguously distinguished in the male by the following diagnostic characteristics, (1) forewings yellowish-brown in dorsal view, clavus with paired large black maculae basally and apically, and (2) pygofer process extending posteriorly farther than apical margin of pygofer, apical one-fifth bent posteroventrally and acute apex, dorsal margin with minute denticles subapically.

##### Key to Chinese species of *Anatkina* Young, 1986 (updated from Yang et al. 2017)

**Table:** 

1	Forewing completely black	***A. candidipes* (Walker)**
–	Forewing not black, or incompletely black	**2**
2	Pronotum unicolorous, without distinct spots or stripes	**3**
–	Pronotum with distinct spots or stripes	**12**
3	Pronotum black	**4**
–	Pronotum not black	**7**
4	Forewing with two distinct red longitudinal stripes, aedeagal shaft with preapical portion wing-shaped produced laterally, apex aviform in ventral view	***A. alata* Yang, Meng & Yu**
–	Forewing with large red, yellow, or orange area, but not two red longitudinal stripes, aedeagal shaft without processes	**5**
5	Forewing large region red, occupying 3/4 length, with one large median black spot; male subgenital plates long triangular, straight, tips sharpened	***A. mediana* Kuoh**
–	Forewing large region red, yellow-brown or orange-yellow, occupying only 1/2 length, without black spot; male subgenital plates curved dorsad, tips blunt	**6**
6	Both sides of head with one pale yellow small spot; face pale yellow, only basal margin and two sides of clypeus black; male pygofer process with two long spine-like processes, connective U-shaped	***A. zhangi* Yang, Meng & Yu**
–	Both sides of head without pale yellow spot; face pale yellow, basal margin, apical margin and two sides of clypeus and basal margin of clypellus black; male pygofer process with only one spine-like process, connective V-shaped	***A. polycolora* Yang (part)**
7	Forewing with one longitudinal red stripe in middle	***A. rufistriata* Yang & Li**
–	Forewing without red stripe	**8**
8	Head with large black spot in middle	**9**
–	Head without large black spot in middle	**11**
9	Crown vertex without black spot, face with U-shaped broad black band centrally	***A. zuoi* Yang & Zhang**
–	Crown vertex with black spot	**10**
10	Crown vertex with two black spot, face without any stripe	***A. yingjiangana* Yang & Li**
–	Crown vertex with one black spot, frontoclypeus with large black macula on each side	***A. maolanensis* Zhong & Yang, sp. nov**.
11	Male pygofer with a large triangular denticle subapically; aedeagus curved hook-like apically, extending distinctly beyond apex of paraphysis while equal in length to it; mid-dorsal surface of paraphysis bearing a flanged connection to aedeagus, with a minute denticle on lateral margin of apical projection	***A. motuoensis* Yang, Meng & Li**
–	Male pygofer without triangular denticle subapically; aedeagus curved but not hook-shaped apically, posteriorly equal in length to the paraphysis while distinctly shorter than the latter overall; mid-dorsal surface of paraphysis lacking articulated connection to aedeagus, apical projection with smooth lateral margin devoid of denticles	***A. vespertinula* (Breddin)**
12	Head and pronotum dark brown with pale yellow or yellow-brown irregular spots	**13**
–	Head and pronotum not colored as above	**18**
13	Style with finger-like process centrally; paraphyses with tridentate lobe apically	***A. insessa* Young**
–	Style without process centrally; paraphyses without dentate lobe apically, tip sharpened or blunt	**14**
14	Aedeagus distinctly longer than paraphyses, median portion of aedeagal shaft with lateral angular process	***A. horishana* (Matsumura)**
–	Aedeagus shorter or as long as paraphyses, median portion of aedeagal shaft without lateral process	**15**
15	Aedeagal shaft distinctly longer than preatrium, surface smooth without serration; pygofer process with slender median process	***A. jocosa* (Walker)**
–	Aedeagal shaft as long as or shorter than preatrium, surface serrate; pygofer process without long process in middle	**16**
16	Tip of pygofer process not hooked, with short projection subapically	***A. infecta* (Distant)**
–	Tip of pygofer process hooked, without projection subapically	**17**
17	Aedeagal shaft straight, pygofer process with reverse hooked process dorsoapically	***A. harpaga* Yang & Li**
–	Aedeagal shaft curved, pygofer process angled apically, and twisted dorsad	***A. attenuata* (Walker)**
18	Forewing with distinct longitudinal red stripes	**19**
–	Forewing without red stripes	**22**
19	Crown with four red spots located on line between ocelli	**20**
–	Crown without red spots	**21**
20	Pronotum with five red longitudinal stripes; thorax and abdomen ventrally yellowish-white. Male pygofer bearing a long semicircular process at apex of ventral margin; pygofer process short, distinctly not reaching apex of pygofer. Aedeagal shaft slightly curved dorsad at midlength, terminating acutely	***A. hopponis* (Matsumura)**
–	Pronotum with three red longitudinal stripes; thorax and abdomen ventrally blackish-brown. Male pygofer bearing only a small inverted denticle at apex of ventral margin; pygofer process long, slightly extending beyond apex of pygofer. Aedeagal shaft gradually curving ventrad, terminating obtusely	***A. taiwanana* Yang, Meng & Li**
21	Pronotum with arched transverse red band on central part, forewing with three longitudinal red stripes	***A. wufengana* Yang & Du**
–	Pronotum without red band, forewing with two longitudinal red stripes	***A. bistriata* Yang & Li**
22	Forewing with distinct spots	**23**
–	Forewing without distinct spots	**35**
23	Forewing spots black	**24**
–	Forewing spots red, yellow, blue, pink etc., but not black	**33**
24	Forewing with two black spots	**25**
–	Forewing with more than two black spots	**26**
25	Crown bearing a black spot centrally at basal margin; scutellum with a black spot at apex	***A. illustris* (Distant) (part)**
–	Crown with a U-shaped black spot extending from basal margin to ocelli; three faint black spots distributed anterior to ocelli; scutellum lacking black spot at apex	***A. tortuosa* Zhong & Yang, sp. nov**.
26	Pronotum with three black spots	**27**
–	Pronotum with more than three black spots	**28**
27	Spots on pronotum arranged in triangle, anterior one small, posterior two big and of equal size	***A. leishanensis* Yang, Meng & Yu**
–	Spots on pronotum arranged horizontally, middle one bigger than lateral ones	***A. billingsi* Young**
28	Caudal margin of male pygofer straight or concave slightly, tip of pygofer process forked into two branches, one short, and another long	***A. nigriventris* Li**
–	Caudal margin of male pygofer round, tip of pygofer process not furcated	**29**
29	Male pygofer process short, not reaching caudal margin of pygofer; subgenital plate with basal 2/3 semicircular	***A. jianfengana* Yang & Li**
–	Male pygofer process long, reaching even beyond caudal margin of pygofer; subgenital plate not semicircular basally	**30**
30	Male pygofer process without process dorsally in middle	***A. incurvata* Kuoh**
–	Male pygofer process with process dorsally in middle, surface of process serrated	**31**
31	Male pygofer process slender, sinuated more than once, tip brush-like, just reaching caudal margin of pygofer	***A. assamensis* (Distant) (part)**
–	Male pygofer process bent only at midlength, apex acuminate and non-penicillate, distinctly extending beyond apex of pygofer in lateral view	**32**
32	Head and thorax dorsally ochraceous-brown; pronotum with black spots often expanded into black stripes. Male pygofer process bearing a short median projection, devoid of triangular lamellate process beneath it; aedeagus sinuate at distal half, style apex snake-head shaped	***A. zhoui* Yang & Li**
–	Head and thorax dorsally orange-red; pronotum with black spots never expanded into stripes. Male pygofer process bearing a long median projection, with a triangular lamellate process beneath it; aedeagal shaft only slightly curved dorsad at midlength, style apex not snake-head shaped	***A. biprocessa* Yang, Meng & Li**
33	Forewing cream yellow, with five red spots	***A. rubromaculata* Kuoh & Zhang**
–	Forewing red, marked with yellow, blue or pink stripes, with less than five red spots	**34**
34	Forewing with three spots, two yellow and one pink	***A. xanthomacula* Kuoh**
–	Forewing with two spots, one blue-white and one pink	***A. livimacula* Yang & Li**
35	Corium of forewing with one black longitudinal stripe	**36**
–	Corium of forewing without black longitudinal stripe	**37**
36	Scutellum black behind transverse depression; forewing red with one longitudinal black stripe	***A. polycolora* Yang (part)**
–	Scutellum yellow-brown behind transverse depression; forewing yellow-brown with two longitudinal black stripes	***A. inflammata* (Distant)**
37	Frontoclypeus with a U-shaped black mark at medioapical portion	**38**
–	Frontoclypeus bearing only a pair of black spots laterally at medioapical portion, devoid of U-shaped connection	**40**
38	Pronotum black, anterior margin orange-yellow; pygofer process furcated apically	***A. rubipennis* Yang & Li**
–	Pronotum orange-yellow with black spots or short stripes; pygofer process not furcated apically	**39**
39	Forewing yellow-brown; pronotum with six black spots arranged in two rows, anterior row two and posterior four, lateral margin carmine	***A. assamensis* (Distant) (part)**
–	Forewing red; pronotum with four transverse black spots arranged in two rows, two anterior and two posterior	***A. fumosa* Kuoh & Zhang**
40	Crown with a pair of black spots at anterior margin; pronotum bearing two black spots on posterior half; frontoclypeus yellowish-white without any dark marks; legs not entirely black	***A. illustris* (Distant) (part)**
–	Crown with a single black spot at anterior margin; pronotum bearing two black spots on anterior half; frontoclypeus with an arrowhead-shaped black mark apically; legs entirely black	***A. nigrilega* Yang, Meng & Li**

## Discussion

This study highlights the limitations of traditional morphological identification methods for Cicadellinae, which primarily rely on external morphology and male genital structures. These limitations are particularly evident in the genus *Anatkina*: species pairs such as *A. attenuata* (Walker, 1851), *A. jocosa* (Walker, 1857), *A. horishana* (Matsumura, 1912) and *A. harpaga* (Yang & Li, 1999) display highly similar external morphologies but distinctly different genital characteristics. Such discrepancies between external morphology and genital characteristics complicate reliable species identification. In addition, the generally indistinct female genital features, combined with intraspecific variation in body size, coloration, and patterning, further hinder accurate identification of female specimens. Integrating molecular systematic data is therefore essential to improve diagnostic precision and to delineate species boundaries more accurately. Future research incorporating biological, ecological, and molecular approaches will be vital for a more scomprehensive understanding of the phylogeny and adaptive evolution of leafhoppers.

## Supplementary Material

XML Treatment for Anatkina

XML Treatment for Anatkina tortuosa

XML Treatment for Anatkina maolanensis

## Additional information

### Use of AI

No use of AI was reported.

### Author ORCIDs

Li-kun Zhong https://orcid.org/0009-0002-2315-3926

Yan Jiang https://orcid.org/0000-0002-8980-5344

Mao-fa Yang https://orcid.org/0000-0002-5523-6825

### Data availability

All of the data that support the findings of this study are available in the main text.
